# Discovering the Potential Mechanisms of Medicinal Mushrooms Antidepressant Activity: A Review

**DOI:** 10.3390/antiox12030623

**Published:** 2023-03-02

**Authors:** Jan Lazur, Kamil Hnatyk, Katarzyna Kała, Katarzyna Sułkowska-Ziaja, Bożena Muszyńska

**Affiliations:** Department of Pharmaceutical Botany, Faculty of Pharmacy, Jagiellonian University Medical College, Medyczna 9 Street, 30-688 Kraków, Poland

**Keywords:** L–tryptophan, psilocybin, BDNF, neurogenesis, neuroinflammation, antioxidant activity, gut–brain axis

## Abstract

Major Depression Disease is a common mental illness that affects more than 322 million people worldwide and it is one of the leading causes of mental and physical disability. The etiology of depression is a complex interplay of psychological, social, and biological factors. Currently, psychopharmacotherapy is based mainly on the monoamine theory, which states that depression is caused by an insufficient level of monoamines such as serotonin, norepinephrine, and/or dopamine. Due to the relatively low efficacy of the typical antidepressant and the high prevalence of treatment-resistant depression (~30%), seeking new ways of prophylaxis, adjuvant therapy, or novel compounds with antidepressant activity, is a priority. According to studies that analyzed mushroom consumption patterns and depression prevalence, it was concluded that mushroom ingestion lowers the odds of depression. Medicinal mushrooms are considered functional foods because of their ability to synthesize and accumulate different types of metabolites, which enhance their health-promoting properties. The review aims to explain the antidepressant activity of edible/medicinal mushrooms by elucidating the mechanism from different perspectives: edible mushrooms as a source of serotonin precursors and psilocybin as a rapid-acting antidepressant. These compounds exhibit anti-neuroinflammatory and antioxidant activities that impact neurotrophin expression, the neurogenesis process, and influence on the gut–brain axis.

## 1. Introduction

As defined by the World Health Organization (WHO), depression is a common mental illness that affects more than 322 million people worldwide [1]. The typical symptoms of depression are persistent sadness, as well as the inability to feel feelings of happiness (anhedonia), sleep problems, loss of appetite, general fatigue, and cognitive problems. Depression is one of the leading causes of mental and physical disability worldwide [2].

The etiology of depression is a complex interplay of psychological, social, and biological factors. People who have experienced adverse life events, such as the death of a loved one or prolonged unemployment, are at an increased risk of developing depression. The presence of physical diseases such as cardiovascular and neurogenerative diseases may increase the risk of depression [3,4].

Interestingly, the COVID-19 pandemic increased depressive symptoms by five percentage points, from 27.8 to 32.8% of adults in the United States in 2021 compared to the first months of 2020 [5]. The increased risk of depressive symptoms and the development of depression may be related to the so-called pandemic environment and the introduction of “lockdowns” that restrict social activities in many countries. There is also growing evidence of COVID-19 disease and an increased risk of depression in recovered adults [6]. The mechanism of the development of depressive symptoms in recovered adults is not well understood yet. One of the possible explanations for this phenomenon may be related to the so-called “cytokine storm”—abnormally high levels of pro-inflammatory cytokines such as IL–1β, IL–6, IL–12, and tumor necrosis factor-alpha (TNF–α) and interferon gamma (INF–γ). A cytokine storm can contribute to neurotoxicity, blood barrier disruption, or even acute necrotizing encephalopathy [7].

Clinical psychopharmacology is a branch of science that deals with the description of the use of pharmacological agents for the treatment of specific psychopathological symptoms. The beginning of this field of knowledge dates to the 1940s and 1950s [8]. The first antidepressant drug was iproniazid, introduced in the treatment of tuberculosis in 1952 [9]. In tuberculosis patients treated with this drug, a significant improvement in mood was observed, unprecedented in patients in this clinical state [10]. After a few years, the mechanism of action of iproniazid was described, as it turned out to be an irreversible inhibitor of the monoamine oxidase (MAO) enzyme, which in turn led to an increase in the concentration of biogenic amines in the brain [11]. Iproniazid became a precursor drug for the first antidepressants, MAO inhibitors, including trancylopromine and phenelzine. Currently, iproniazide is not registered for the treatment of depression due to side effects, including liver damage [11].

Treatment of depression is based on the theory of monoamines introduced in the 1960s, which states that this disease is caused by a decreased level of monoamines (serotonin, norepinephrine, and dopamine) in the brain [12]. The mechanism of action of drugs used in the first line of depression treatment is inhibition of neuronal reuptake of monoamines from synaptic clefts, as in the case of selective serotonin reuptake inhibitors (SSRIs), for example, fluoxetine, citalopram, or sertraline, which reduce the activity of serotonin transporters [13]. Although these drugs are potent antidepressants, the cause of depression is not simply insufficient monoamine levels. SSRIs cause an immediate increase in serotonin transmission, while it takes several weeks for mood-elevating activity to develop in treated patients, which is associated with changes in the expression of serotonin-dependent receptors. Recent data on the development of depression have extended the theory of monoamines to include neurotrophic and neurogenic hypotheses [14,15]. Decreased levels of brain-derived neurotrophic factor (BDNF) are involved in the pathogenesis of depression [16]. BDNF is required for neurogenesis and neuroplasticity in the hippocampus [17]. In people with depression, BDNF expression is decreased in the limbic area of the brain due to neuronal atrophy. Serotonin and its receptors are involved in the regulation of BDNF levels and neurogenesis in the adult hippocampus. Chronic treatment with an SSRI has been shown to increase BDNF levels in humans and rodents [18,19]. Altered levels of other neurotrophins, such as neurotrophin–3 (NT–3), neurotrophin–4 (NT–4), and nerve growth factor (NGF), are also observed in patients with depressive disorders [20].

Medicinal/edible mushrooms and their mycelia from in vitro cultures are receiving increasing scientific attention for their potential to promote health. They are considered functional foods because of their ability to synthesize and accumulate different types of metabolites, which enhance their health-promoting properties and can be used as a supplement to the human diet. Studies show the multidirectional activity of medicinal mushrooms and their mycelium, including antioxidant, anticancer, anti-inflammatory, and immunostimulatory effects. Increasingly, there is also evidence of antidepressant activity [21,22,23].

Researchers at Penn State University published a research paper describing the link between eating mushrooms and depression [24]. The main conclusion of this population-based study, which analyzed mushroom consumption among US residents from 2005 to 2016, was that mushroom consumers are less likely to suffer from depression [24]. The results are consistent with previous small clinical studies [25,26,27]. However, the studies presented above did not investigate the potential mechanisms of the antidepressant effect of edible mushrooms.

This review aims to explain the antidepressant activity of edible/medicinal mushrooms by elucidating mechanisms from different perspectives, starting with answering the question of whether edible mushrooms can be a good source of indole compounds, such as L–tryptophan (Trp)—a precursor of the brain serotonin synthesis pathway. Although psilocybin-containing mushrooms are considered inedible, psilocybin and its active constituent, psilocin, is a real candidate for being classified as a rapid-acting antidepressant (RAAD) which is especially important for patients suffering from treatment-resistant depression (TRD). That is why this review also describes the current knowledge about the mechanism of action of psilocybin action and summarizes the current progress of clinical trials considering the usage of psilocybin in TRD. The review summarizes the anti-inflammatory effect of the administration of edible/medicinal mushrooms in alleviating neuroinflammation and the influence of analysis on the activity of the kynurenic pathway for in vitro and in vivo models. Furthermore, the neurotrophic and neurogenic activity of selected edible/medicinal mushrooms of in vitro and in vivo models was summarized. The last part of this review focuses on summarizing the current knowledge of edible/medicinal mushroom species—extracts or isolated substances on the gut microbiota, which has been extensively studied over the last five years.

## 2. L–Tryptophan Derivatives—Essential Compounds for Serotonin Synthesis

L–Tryptophan (Trp) and its derivatives, such as 5-hydroxy–L–tryptophan (5-OH-L-Trp), and tryptamine, are related to biochemical reactions that lead to serotonin synthesis in the brain’s neurotransmitters, lower levels of which are observed in clinically depressed patients [28] These compounds have been shown to scavenge free radicals and protect cells against oxidative stress, potentially reducing the risk of certain diseases such as cancer, neurogenerative diseases, and depression [29].

**Figure 1:**
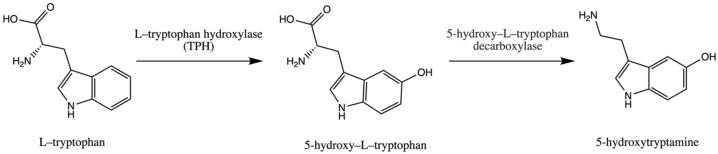
Pathway of serotonin (5–hydroxytryptamine) synthesis.

Trp is an essential amino acid and is considered an exogenic amino acid for the human body. Although its importance is the synthesis of various proteins, Trp is a precursor of serotonin (5-hydroxytryptamine) in the brain and gut. The biosynthetic pathway of serotonin is presented in Figure 1.

The serotonin metabolic pathway starts with the hydroxylation of Trp to 5-OH-L-Trp, which is decarboxylated to 5-hydroxytryptamine (serotonin). The limiting stage of serotonin synthesis is Trp hydroxylation by the enzyme Trp hydroxylase (TPH) and is not saturated at physiological brain tryptophan concentrations; therefore, serotonin synthesis in the brain is assumed to be directly connected with tryptophan transport into the brain [30,31,32].

Trp can be transported to the brain through a nutrient amino acid transporter protein that is involved in the transport of large neutral amino acids (LNAAs) such as valine, leucine, isoleucine, tyrosine, phenylalanine, and methionine from the bloodstream to the brain through the blood-brain barrier (BBB) [33]. The content of Trp that crosses the BBB by the nutrient amino acid transporter depends on the ratio of Trp and other LNAAs in plasma [33]. After meal ingestion, the levels of Trp and other LNAAs in plasma increase. As a result of a relatively low increase in Trp in comparison to other essential amino acids in plasma concentration, the plasma Trp/LNAA ratio decreases, and consequently, a reduced Trp influx to the brain is observed [33].

There are several factors that can influence Trp influx to the brain by influencing LNAA concentration in plasma, such as the ingestion of carbohydrates, the intake of protein amounts, or exercise. Ingestion of dietary carbohydrates led to elevated insulin levels. Insulin promotes the uptake of LNAAs in skeletal muscle, which leads to an increase in the Trp/LNAA ratio and consequently to Trp influx into brain tissue [34]. L–Tryptophan is not transported to muscle tissue because it bonds with albumin, while other LNAAs are not.

Trp obtained from food can be transformed into serotonin in a limited amount. In mammals, approximately 95% of Trp is metabolized through the kynurenic metabolic pathway, whose products exhibit biological activity [35].

Fruiting bodies of edible mushrooms are a good source of non-hallucinogenic indole compounds such as Trp, 5-OH-L-Trp, and tryptamine (Table 1) [36,37,38].

The highest content of Trp and its hydroxylated derivative was observed in *Pleurotus djamor* (respectively, 24.84 and 193.95 mg/100 g dw) and *Suillus bovinus* (respectively, 25.9 and 15.83 mg/100 g dw) [39].

According to scientific data, nonfungal sources that contain high levels of Trp include soy seeds (680 mg/100 g dw), pumpkin seeds (580 mg/100 g dw), and spirulina (930 mg/100 g dw) [45]. Other researchers described that transgenic soybean plants were found to accumulate Trp at levels as high as 380 to 480 mg/100 g dw of seed flour, up to a 12-fold increase compared to Trp levels in non-transgenic seeds [46]. Wheat—durum 169 mg/100 g dw, rye 125 mg/100 g dw, barley 165 mg/100 g dw, chickpea 220 mg/100 g dw, lentil—red 139 mg/100 g dw, and kidney beans 240 mg/100 g dw are also considered good natural sources of Trp [45]. In contrast, the seeds of the *Griffonia simplicifolia* plant are considered one of the best natural sources of 5-OH-L-Trp [47,48]. Its content can be as high as 156 mg/g dw (16% of the seed weight) [48]. However, Maffei points out that mushrooms can also be a good source of this substance [47]. When analyzing the plant sources of Trp, it turns out that tomatoes are a good source (14.71 mg/100 g dw), while smaller amounts were determined in strawberries (5.7 mg/100 g dw), lettuce (2.5 mg/100 g dw), spinach (0.65 mg/100 g dw) or chicory (0.08 mg/100 g dw) [49]. Thus, it appears that both mycelium and fruiting bodies can provide an alternative source of Trp and Trp derivatives. The content of biologically active substances in mushroom samples is mainly measured after extracting them with various solvents such as methanol and ethanol from raw, lyophilized fruiting bodies. To determine the usage of selected edible mushrooms as sources of indole compounds, the influence of various types of thermal preparation of edible mushrooms on the content of biological active substances was analyzed [40,41]. Thermal processing (dry material suspended in water and thermostated at 100 °C for 60 min in a Soxhlet apparatus) was shown to result in approximately 2 times lower indole compound content after thermal processing compared to the unprocessed. However, Trp content increases relatively in processed samples compared to that of unprocessed one. The increase in Trp content can be explained by the fact that 5-OH-L-Trp or serotonin degradation at higher temperatures [40]. The results were confirmed in another study [41]. In conclusion, the method of preparing meals with mushrooms can affect the content of indole compounds because of their sensitivity to elevated temperature. However, thermally processed mushrooms remain a good source of Trp and 5-OH-L-Trp [40,41].

Today, dietary supplements containing lyophilized fruiting bodies, extracts, or even mycelium from edible mushroom species are available in community pharmacies or in stores with so-called healthy food. Mycelium can be obtained through in vitro cultures initiated from specially prepared parts of the fruiting body, the hymenial area. One of the most important advantages of in vitro cultures is the fact that the content of biological active substances does not differ between batches because the condition of the in vitro culture is monitored and maintained at specific parameters depending on the mushroom species. Studies have shown that the content of indole compounds in biomass from in vitro cultures can be much higher than that in fruiting bodies [50]. The content of selected indole compounds in mycelia from in vitro cultures is presented in Table 2.

In most cases, the content of Trp is higher in mycelia than in the fruiting bodies of selected edible mushroom species, especially *Pleurotus citrinopileatus* and *Pleurotus djamor*. The most notable change can be observed in the content of 5-OH-L-Trp, which is almost four times higher in the mycelium compared to the fruiting bodies [39]. Another advantage of making mycelium from edible mushroom species a dietary supplement is that powdered mycelium or mycelial extract does not have to be thermally processed, so thermolabile substances will not degrade.

Modification of the composition of the in vitro medium, such as the addition of indole precursors—anthranilic acid and serine—can have a positive influence on the content of indole compounds in mycelia [55]. In one experimental study, in vitro culture medium was supplemented with various concentrations of serine or anthranilic acid (0.1–0.75 g/L). For in vitro cultures of *A. bisporus* and *I. badia*, the most optimal precursor concentration was 0.5 g/L of serine for *A. bisporus* or 0.5 g/L of anthranilic acid for both species analyzed according to the content of indole compounds. The addition of 0.5 g/L of serine to *A. bisporus* in vitro cultures resulted in the highest total concentration of indole compounds (186.37 mg/100 g dw). The addition of 0.5 g/L anthranilic acid to in vitro cultures of *I. badia* and *A. bisporus* resulted in the highest total concentration of indole compounds, 352.06 dw and >200 mg/100 g dw [55].

The liberation of biological substances, which is the number of substances released from the matrix of food or dietary supplement formula (tablets, hard capsules), can be measured in vitro using models of the human gastrointestinal tract. Therefore, only substances free of their matrix can be absorbed in the gastrointestinal tract. The analysis of the liberation of indole compounds was performed for *Agaricus bisporus* mycelia [56]. In the study, the content of indole compounds in artificial gastric and intestinal juice was measured after 5 time points—15, 30, 60, 90, and 120 min of incubation. The highest 5-OH-L-Trp content was established between 91.99 and 324.64 mg/100 g dw after 30 min of digestion in artificial gastric juice and after 150 min of incubation in artificial intestinal juice [56]. In a similar study on the release of indole compounds from fruiting bodies and *Tricholoma equestre* mycelia, 5-OH-L-Trp was released in the highest amount from freeze-dried mycelia after 120 min of incubation in artificial gastric juice (352.47 mg/100 g dw) and after 15 min of incubation in artificial gastric juice in the case of fruiting bodies (281.56 mg/100 g dw) [57]. For the fruiting bodies of *Suilius bovinus*, the highest content of released 5-OH-L-Trp was observed after 120 min of incubation in artificial gastric juice (237 mg/100 g dw) (for liberation study of *Imleria badia*, *Boletus edulis*, *Cantharellus cibarius*, *Lactarius deliciosus*, *Leccinum scabrum*, *Armillaria mellea*, *Suillus luteus*, *Pleurotus ostreatus*, *Auricularia polytricha*, see [58]). Based on the studies mentioned above, it can be concluded that indole compounds are released in the highest amount in artificial gastric juice compared to artificial intestinal juice. Trp is not readily liberated, regardless of whether it is from fruiting bodies or mycelia from in vitro cultures. However, 5-OH-L-Trp was one of the indole compounds that was released at the highest amount, regardless of the species analyzed [56,57,58].

Another important factor that should be considered when fruiting bodies or mycelium are thought to be a source of indole compounds is the bioavailability of these compounds. Bioavailability is a term used to describe the percentage or amount of a xenobiotic that reaches the systemic circulation [59]. In the case of the bioavailability analysis of secondary metabolites such as indole compounds, it will be the amount of indole compound that reaches the systemic circulation. The evaluation of the bioavailability of natural compounds in humans is rare due to requirements and restrictions imposed by ethics commissions, therefore, alternative methods involving, for example, the colon epithelial cells (CaCo-2) cell line are used to estimate the bioavailability of active substances [60]. In the study of indole absorption from *Imleria badia* mycelia, the CaCo-2 cell line was used to measure active transport, while semi-permeable membranes were used in the passive transport model after release of biological active substances in the human gastrointestinal tract model. The bioavailability of 5–hydroxy–L–tryptophan ranged from 5.21 to 11.92% using active transport modes (depending on mycelial in vitro culture conditions—an addition of zinc (VI) sulfate or zinc hydrogen aspartate). Through the passive transport model, 5–hydroxy–L–tryptophan accounted for 2% of the compound released into artificial digestive juices [61]

## 3. Tryptamine Derivatives—Psilocybin as a Potential Rapid Acting Antidepressant

Among patients with Major Depressive Disorder (MDD), almost 30% suffer from a treatment-resistant one [62]. To date, there is no one definition of TRD, but the most common criteria found in the literature are: failure to respond to at least two antidepressants with different mechanisms of action treatment, confirmation of adequate dosage, and duration of treatment longer than 4 weeks for each antidepressant without effect [63].

Patients suffering from TRD or for those for whom antidepressant action should be obtained in a shorter time compared to conventional — for example, patients at high risk of suicide may benefit from a novel group of antidepressants, rapid-acting antidepressants (RAADs) [64].

The definition of RAADs has not yet been specified, but contrary to conventional antidepressants, which require a few weeks to produce significant antidepressant action, they need one or a few doses to produce a significant impact on depressive symptoms or even remission, especially in a group of patients who did not respond to first-line treatment [65]. There are several mechanisms that can be responsible for the rapid antidepressant action of some drug candidates, for example, NMDA receptor antagonism, muscarinic receptors, and classic psychedelic drugs such as psilocybin or LSD, which influence serotonergic activity. The first drug registered for the TRD with a rapid-acting mechanism is esketamine, the ketamine enantiomer, approved by the US Food and Drug Administration and the European Medicines Agency in the form of a nasal spray called Spravato^®^ [66]. Esketamine is a non-competitive NMDA receptor antagonist. It selectively induces an antagonist effect in the event of excessive activation of NMDA that leads to an increased concentration of extrasynaptic glutamate and activation of neuroplasticity pathways [67]. The high effectiveness of esketamine in the treatment of TRD leads researchers to seek other candidates for RAADs such as psilocybin.

Psylocibin (3-[2-(dimethylamino)ethyl]-1H-indol-4-yl dihydrogen phosphate) is a natural substance which is a secondary metabolite found in the following genera: *Psilocybe, Copelandia*, *Pluteus*, *Gymnopilus*, *Pholiotina*, *Galerina*, *Inocybe* [68,69,70,71,72,73]. It is not biologically active and must be dephosphorylated to become psilocin, a psychoactive compound. Psilocin, as a classic psychedelic, has an agonist or partially agonist effect on 5-HT_2A_ receptors, which is a possible explanation of the hallucinogenic effect of this compound as these receptors are highly expressed in the visual cortex [74]. The following theory was confirmed by the administration of a selective 5-HT_2A_ antagonist, ketanserin, to humans, which attenuated the hallucinatory effect of psilocybin [75].

Administration of psilocybin induces down-regulation of 5-HT_2A_ receptors, that overexpression is observed in patients with major depression disorder [76,77]. Another theory postulates that downregulation of 5-HT_2A_ receptors may be influenced by increased synthesis of BDNF in the medial prefrontal cortex (mPFC) after administration of psilocybin. Elevated levels of BDNF can be explained by modulation of AMPA and NMDA receptors through the effect of psilocybin administration as an agonist to 5-HT_2A_ receptors [78]. Activation of this receptor has a positive influence on cerebral neuroplasticity by increasing BDNF synthesis and increasing c-FOS factor expression in the anterior cingulate cortex and mPFC—areas of the brain implicated in depression [79]. The secondary mechanism of antidepressant action is its anti-inflammatory effect of psilocybin by decreasing levels of TNF–α and IL–1β, which was demonstrated in the human U937 macrophage cell line by administering psilocybin-containing mushrooms’ water extract [80]. An increase in the level of pro-inflammatory cytokines, such as TNF-α, is one of the causes of the activation of the kynurenic pathway in microglia and the production of neurotoxic compounds, hydroxykynurenine and quinolinic acid [81].

The use of psychedelics in the treatment of MDD has gained scientific attention due to the relatively low therapeutic effectiveness of current psychopharmacological approaches and the increasing knowledge of the pathophysiology of depression. Recent meta-analysis of psychedelic therapy for depressive symptoms carried out by the Ko team revealed that the definitive clinical efficacy of the use of psychedelics such as psylocybin, LSD, or ayahuasca for depressive symptoms has not been demonstrated, partly due to the lack of a sufficient number of randomized clinical trials [82]. The largest clinical trial on the use of psylocibin in treatment-resistant depression was carried out by the Goodwin team with participants from ten countries in Europe and North America. The trial consisted of 233 participants, divided into three groups that were given a single dose of 25, 10, and 1 mg (control) of psilocybin. The change in value of the Montogomery-Åsberg Depression Rating Scale, the tool that is used to stratify the severity of depressive episodes in adults, from baseline to week 3 was the primary endpoint of the trial. Psilocybin at a single dose of 25 mg reduced depression scores significantly more than a 1-mg dose over a period of 3 weeks, but was associated with adverse effects such as headache, nausea, and dizziness [83]. Although there is no definitive verdict on the use of psychedelics in the treatment of Major Depressive Disorder, based on randomized clinical trials conducted with psilocybin, short- and long-term reductions in depressive symptoms have been observed [83].

Gotvaldová and her team performed a quantitative analysis of tryptamine derivatives in fruiting bodies of genera such as *Psilocybe*, *Pluteus*, and *Inocybe* [84]. The highest content of psilocybin in the analyzed species of *Psilocybe* genra was in *Pmexicana* (3.29–3.93 mg/g dw), *P. caerulipes* (2.23–5.67 mg/g dw), *P. cyanescens* (2.34–13.8 mg/d gw), and *P. serbica var. moravica* (5.65–14.16 mg/g dw). The highest concentration of psilocybin in *Pluteus* genra was found in *P. americanus* (1.17–2.43 mg/g dw). In *Inocybe* genra, the amount of psilocybin did not exceed 0.282 mg/g dw (*I. corydalina*) [84]. Psilocybin is not present in edible mushrooms; concentration of this compound in *Agaricus bisporus* was lower than the limit of detection [84]. 

Few biotechnological attempts have been made to increase the content of tryptamine derivatives in fruiting bodies or mycelia in mushroom species containing psilocybin [85,86]. The most interesting biotechnological method for obtaining psilocybin is the production of this compound, which was proposed by the Milne team using metabolically engineered yeast (*Saccharomyces cerevisiae*), whose productivity was determined at 120.3 mg/L of psilocybin. Due to further modification of the transformed metabolic pathway with *P. cubensis* cytochrome P450 reductase, it was possible to obtain a production of psilocybin of 627 mg/L, which allows a relatively cheap production of psilocybin on an industrial scale [85]. The highest production of psilocybin was observed in genetically modified *Escherichia coli* in which 1.16 g/L was observed through the biotransformation of 4–hydroxindole, serine, and methionine. However, the method would be difficult to implement in an industrial setting because of the high price of substrates [86].

Microdosing is the practice of repeatedly using low doses of psychedelics such as psilocybin. It is believed that the consumption of low doses of psilocybin (around 0.5 g per dose) can improve cognitive performance, stimulate creativity, and increase stamina. Microdoses do not induce hallucinations, contrary to the regular dose used for recreational use [87]. Possession or consumption of psychedelics such as LSD or psilocybin is illegal in most countries, but there is increasing evidence of the use of microdoses of psychedelics. What is the main motivation for the consumption of “magic mushrooms”? In the survey that collected the responses from 1116 respondents through an online questionnaire, it was found that the main motivations for microdosing psychedelics were performance improvement (37%), mood improvement (29%), and curiosity (15%) [88]. The consumption of microdoses of psychedelics is considered safe by those who decide to try it for the first time, but it showed that almost 20% of consumers experienced some acute psychological or physical negative effects [88]. There are more studies describing microdosing phenomena among humans, but due to their type (online surveys, observation, and open-label studies). the results are prone to confirmatory bias, as many lack a control group or are based on self-selected samples [88,89,90]. Some respondents reported a positive effect of microdosing on their cognitive abilities and creativity. However, it should be considered that in most cases, this may be due to the approach associated with the expectation of the positive effects of respondents and the researchers themselves conducting the observational study. A double-blind, placebo-controlled study of psylocybin microdosing carried out with 35 participants revealed no evidence to support enhanced cognitive or creative function. Low doses of psilocybin (0.5 g of dried fruiting bodies of *P. cubensis* two times a week, the total dose equals to 1.0 g) even resulted in small cognitive impairments [91].

So far, the results of clinical trials on the use of psilocybin in controlled clinical conditions give hope to patients suffering from TRD. In contrast to the antidepressants currently used, their effect appears several hours after the first dose of the preparation and lasts several days, which means that the total number of doses is lower than in the case of drugs administered every day, which can positively affect adherence to medical recommendations. More clinical trials are needed before psilocybin can be approved for the treatment of patients with TRD, especially those evaluating long-term antidepressant effects.

## 4. Anti-Inflammatory Activity of Medicinal Mushrooms in Beating Depression

The link between immune system, inflammation and depression was observed for the first time when IFN–α therapies, which activate inflammatory antiviral response, were introduced as a treatment for hepatitis C. Patients treated with interferon developed depression-like behaviors after 4 weeks of treatment initiationinitiation [92]. Patients with MDD have been observed to have higher levels of pro-inflammatory mediators such as IL–6, IL–12 and C–reactive protein compared to nondepressed individuals [93,94]. Patients with TRD are more likely to have elevated pro-inflammatory markers [95].

Peripheral inflammation can affect the central nervous system in many ways. It starts by having a negative impact on the permeability of the blood-brain barrier, which makes cytokines and immune cells more likely to cross to the brain [96]. The possible crossing of the proinflammatory cytokines to the brain may alter the kynurenic pathways, which are correlated with tryptophan availability.

The activity of enzymes involved in the kynurenic pathway can be modulated by glucocorticosteroids and/or pro-inflammatory cytokines. The hypothesis of depression induction caused by tryptophan depletion in the brain was first stated by Fuch et al. in 2002 [97]. However, research carried out by Dunn and Welch demonstrated that administration of LPS and/or proinflammatory cytokine IL–1 to mice increases brain tryptophan and serotonin concentration [98]. The O’Connor team conducted a similar observation that showed that LPS administered to rodents resulted in increased kynurenine content in the brains of mice and brain tryptophan and serotonin [99]. In human studies, patients treated with IFN–α showed that tryptophan concentrations in cerebrospinal fluid were stable despite a decrease in Trp blood level [100].

Based on the observations mentioned above, it is unlikely that depressive symptoms can be caused or worsened by tryptophan depletion in the brain by shifting tryptophan to the kynurenic pathway. An alternate hypothesis is that alterations in concentrations of products of the kynurenine pathway may play a role in the development of depression [81,101].

Kynurenine can be metabolized in several ways, depending on the cell type in which kynurenine is produced, transported, and metabolized. In microglia, kynurenine is broken down to 3–hydroxykynurenine and quinolinic acid, which are neurotoxic. Neurotoxicity of these compounds is caused by the generation of reactive oxygen species that may damage neural cells and act as agonists in the NMDA receptor [102]. Reactive oxygen species can promote the production of proinflammatory cytokines via NF-κB pathway [103]. In astrocytes, kynurenine is degraded to kynurenic acid knows from its neuroprotective activity by acting as an antagonist of the NMDA and alpha-7 nicotinic acetylochine receptor [104]. The intact neuron can metabolize kynurenine to picolinic acid, which is also neuroprotective.

In patients with depression, variations in the levels of the products of the kynurenic pathway have been observed. In a meta-analysis carried out by Ogyu et al. it was observed that in depressed patients decreased level of kynurenine and kynurenic acid was observed whereas depression free patients were observed with higher level of quinolic acid [105].

Neuroinflammation may be beneficial because activation of microglia is necessary to eliminate the threat in the form of infection, injury, or toxic metabolites [102]. Although chronic neuroinflammation can lead to overproduction of pro-inflammatory cytokines and production of neurotoxins (products of the kynurenic pathway described above) that can lead to neuronal death and, consequently, loss of neuronal volume in areas responsible for mood regulation such as the PFC or the hippocampus. Additionally, neuroinflammation may be an important part of the pattern of neurodegenerative diseases such as Alzheimer’s and Parkinson’s disease [106]. In vitro and in vivo studies showing the anti-inflammatory activity are presented in Table 3. 

Research on potential anti-neuroinflammatory activity is based on activity analysis of TLR4 and NF-κB pathways in cells in order to observe whether the addition of investigated mushroom species extract/isolated substance exhibits this activity.

Toll-like receptors (TLRs) are a group of transmembrane receptors responsible for the recognition of pathogen-associated molecular patterns (PAMPs) or danger-associated molecular patterns (DAMPs). The activation of TLR4 receptor by bacterial lipopolysaccharide (LPS), viral proteins and polysaccharides results in the production of inflammatory substances, which are essential in order to produce effective immune response [123]. Activation of TLR4 leads to activation of the nuclear factor kappa-light-chain-enhancer of activated B cells (NF-κB). NF-κB is one of the most important and versatile family of transcriptional factors which are associated with inflammation and immunity [124]. After stimulation, NF-κB factors complex are being freed and in free form (p50 and p65) translocate to nucleus which activate transcription and liberation of pro-inflammatory mediator, such as inducible nitric oxide synthase (iNOS) and NO, prostaglandin E2 (PGE2) and cyclooxygenase–2 (COX-2) and proinflammatory cytokines such as IFN-γ, IL-1β, IL-6, and TNF-α [124].

## 5. Medicinal Mushrooms and Their Impact on Neurotrophins—Neurotrophin and Neurogenesis-Based Depression

The neurogenesis hypothesis of depression postulates that depressive behaviors can be attributed to insufficient or altered production and maturation of new neurons—adult neurogenesis [125,126]. The reduced volume of the hippocampus and mPFC is one of the most described neural abnormalities in depressed patients. However, it is still not concluded whether depression is a cause of neuronal atrophy or whether neuronal atrophy is a cause of depression. Conclusions from observation of hippocampus and mPFC volumes in first-onset MDD are not conclusive [89]. A meta-analysis revealed that a reduction in hippocampal volume is observed in patients with a history of MDD of at least two years [127]. Currently, there are three different approaches according to the cause versus consequence debate on the reduction of hippocampal and mPFC volume in depressed patients [128]. The causative one postulates that reduced mPFC and hippocampal-volume cause initial depression, which is supported by human studies that showed that patients with reduced hippocampal and mPFC volume have a have a higher chance of onset of MDD [129,130]. In the opposite approach, the reduced volume of the hippocampus and mPFC is caused by multiple episodes of depression—the hypothesis is supported by longitudinal studies that showed that the decreased volume of structures is more obvious when MDD does not relapse [131,132]. The third approach is that reduced volumes of mPFC and hippocampus do not have to be related to MDD itself, as there are some studies showing reduced volumes of these structures in healthy subjects exposed to chronic stress in life [133].

The proteins involved in the regulation of cell proliferation, maintaining synaptic plasticity, and neural functions are neurotrophins, such as NGF, NT-3 and NT-4 [134]. Brain-derived neurotrophic factors are the most studied member of the NGF family. In general, neurotrophins are synthesized as proneurotrophins that are processed intra- or extracellularly to be secreted in a mature and biologically active form. Proneurotrophins react with the p75 neurotrophin receptor (p75^NTR^) and thus mediate neuronal death, leading to decreased synaptic plasticity, while mature neurotrophins bind to a particular tyrosine kinase receptor (Trk), leading to promotion of survival and differentiation by increasing the branching of axons and dendrites [135,136,137,138,139]. Clinical evidence supporting the neurotrophic hypothesis of depression is based on postmortem studies that demonstrated that BDNF levels are decreased in the cerebral cortex of depressed and suicide subjects [140,141]. Studies confirmed that in depressed patients, a reduced volume of the PFC and hippocampus was observed, which can be explained by a decrease in the signaling of BDNF-TrkB [142,143,144]. It was suggested, in a very simplified way, that an increased level of mature BDNF through TrkB signaling may produce an ‘antidepressant’ response, while pro-BDNF through p75^NTR^ signaling may exhibit the opposite effect [145].

There is research on the impact of the extract from the fruiting bodies or mycelia or isolated substances from medicinal/edible mushroom species on the survival of neuron cells in in vitro and in vivo models and analyses of the influence on neurotrophin expression—BDNF, particularly.

An antidepressant activity of polysaccharide–peptide (PGL) isolated from *Ganoderma lucidum* spores was measured in mice. PGL reversed depression behaviors in mice after acute and chronic administration. The administration of isolated PGL resulted in upregulation of BDNF/TrkB expression in the prefrontal cortex [146].

*Hericium erinaceus* (Lion’s mane) is one of the most investigated medicinal mushroom species in terms of antidepressant activity. The popularity of this species is particularly caused by the presence of erinacines, a group of biologically active substances that is a stimulator of NGF [147].

Erinacines are chemical components that can be classified as cyathin diterpenoids that are found in the mycelium of *H. erinaceus* but not in fruiting bodies. To date, 15 erinacines have been isolated and described—the neuroprotective effect shows erinacine A–I [148]. In a study in rats with induced ischemic brain injury, administration of mycelium or isolated erinacine A resulted in inhibition of neuronal cell death. Erinacine A acts as a reactive oxygen species scavenger and inhibitor of the iNOS/p38 MAPK and CHOP proteins, which protect neurons from death caused by ischemic injury. In addition, the increase in pro-inflammatory cytokine levels observed in the control group after ischemic injury was reversed after administration of erinacine A in a dose-dependent manner [149]. In another study, a standardized aqueous extract of *H. erinaceus* was administered to male C57BL/6 mice subjected to chronic restraint stress for 4 weeks. Upregulation of mRNA and increased expression of proteins related to neurogenesis such as BDNF, doublecortin, nestin, synaptophysin, tropomyosin receptor kinase B (TrkB) were observed in mice fed *H. erinaceus* extract. Furthermore, bromodeoxyuridine positive cells were observed in the hippocampus, indicating enhanced neurogenesis [150]. Neurotrophic group of substances isolated from the fruiting bodies of *H. erinaceus*, isoindolinones—hericerin, isohericerinol A, N-de-phenylethyl isohericerin and corallocin A. Increased NGF production in C6 glioma cells was observed. Increased expression of NGF, BDNF, and synaptophysin (SYP) was observed in the C6-N2a cell line [151].

Biological activity and safety data obtained in the course of many in vitro and in vivo studies of *H. erinaceus* led to the conducting of a few clinical trials in humans. The first randomized, double-blind, placebo-controlled clinical trial was conducted in a small group of 30 women, who in the experimental group ingested powdered fruiting bodies of *H. erinaceus* in the form of cookies. Based on the result comparison of the Center for Epidemiologic Studies Depression Scale (CES-D) and the Indefinite Complaints Index (ICI), it can be concluded that *H. erinaceus* ingestion for at least 4 weeks (0.5 g of powdered fruiting bodies) may result in reduced depression-like symptoms and anxiety [152]. In a more recent clinical trial, whose objective was to investigate the effect of *H. erinaceus* on hearing degeneration in elderly patients (*n* = 80), it was proved that administration of *H. erinaceus* mycelium could reduce hearing loss, especially for high frequencies and speech recognition. *H. erinaceus* administration promoted the concentrations of BDNF and NGF in patients over 65 years old better than in younger patients [153].

Another species of the *Hericium* genra, *Hericium coralloides*, is a source of the benzofuranone and isoindolinone structure groups—corralocins A–C. The greatest impact on the stimulation of neurite outgrowth from PC12 cells was observed in the case of the addition of corralocin C to the culture medium—the same compound was shown to be the NGF—inducer in the 1321N1 astrocyte cell line. The expression of BDNF mRNA was evaluated in 1321N1 astrocytes after stimulation with corralocins A–C. The highest level of BDNF mRNA was observed after corralocin C stimulation [154].

2α-hydroxy-inotodiol (2α-HI)—a lanostane tripterpenoid isolated from *Inonotus obliquus* exhibited the most remarkable neuroprotective activity among 10 isolated structures from this species. Neuroprotective activity was measured in vitro in H_2_O_2_-induced SH–SY5Y cells. 2α-HI showed neuroprotective effects through activation of Nrg2 and BDNF/TrkB/ERK/CREB pathway. The result of the in vitro study was confirmed in vivo in zebrafish [155].

The 28 kDa polysaccharide peptide isolated from *Ganoderma lucidum* spores showed antidepressant activity in mouse model of depression induced by unpredictable chronic mild stress. The antidepressant mechanism of action was the upregulation of BDNF in the PFC [146].

Polyoxygenated cyanthane diterpenoids, in addition to their anti-neuroinflammatory activity, showed neurogenesis inducing activity by NGF–induced neurite growth activity in PC–12 cells after the addition of isolated diterpenoids in all concentrations tested [110].

β–glucans depending on their chemical structure, may have different effects on cognition. The difference in biological activity of three β–glucans from mushroom (β-(1,3)/(1,6)-glucan isolated from *L. edodes*), curdlan (β-(1,3)-glucan) and oat bran (β-(1,3)/(1,4)-glucan) was evaluated in animal model (C57BL/6J mice) [156]. All three glucans had a positive effect on temporal order recognition memory. The expression of BDNF and postsynaptic protein 95 increased in the PFC. In addition, only in supplementation with mushroom β–glucan, post-synaptic thickness of synaptic ultrastructure was observed [156]. Lentinan, an β-(1,3)/(1,6)-glucan polysaccharide found in *L. edodes* inhibited neuroinflammation and enhanced remyelination in the cuprizone (CPZ) mouse model [108]. Lentinan reversed neuronal injury as well as motor dysfunctions through dectin-1 receptor. It shows that lentinan could be a novel therapeutic agent that can reduce demyelination, which is present in multiple sclerosis [108].

## 6. Medicinal Mushroom and the Brain–Gut Axis

The human gut microbiota has become the subject of intensive research in recent years. The brain–gut axis refers to the bilateral link between the intestinal microbiome and the central nervous system. The gut microbiota plays an important role in the processing of carbohydrates, the production of some vitamins (B group vitamins and vitamin K), or the synthetization of short-chain fatty acids (SCFAs).

The gut microbiota also plays a crucial role in modulating the pharmacokinetic parameters of some drugs, including those that act on the CNS. The presence of *Helicobacter pylori* can induce a reduction in L-dopa absorption, which can have a negative impact on the time of onset, duration, and quality of life parameters in patients with Alzheimer’s disease [157]. The presence of *Clostridium leptum* in the gastrointestinal tract of humans may affect nitrazepam metabolism due to the presence of the nitroreductase enzyme. As a result, 7–aminonitrazepam is produced, which is known for its toxic activity [158]. Similar observations were made in the biotransformation of clonazepam to 7-aminoclonazepam, leading to a higher risk of toxicity [159]. In another study, the presence of *Clostridium sporogenes* and the reductase activity found in this strain altered the metabolism of zonisamide—an anticonvulsant leading to increased drug activity [160].

In addition to the possible interaction between the gut microbiota and drugs that can result in suboptimal drug activity, altered gut microbiota composition may be one of the pathomechanisms that explain some neurodegenerative diseases such as Alzheimer’s disease, Parkinson’s disease, multiple sclerosis, MDD, or diseases with the predominant symptom of anxiety [161]. The connection between the gut microbiota and depression may be attributed to an inflammation-based theory: alteration of the gut microbiota in a direction favorable growth of pro-inflammatory taxa such as *Enterobacteriaceae*, *Eggerthella*, *Desulfovibrio* may contribute to the initiation of inflammation [162,163,164]. Increased pro-inflammatory mediators can interfere with the blood-brain barrier and cause inflammation, degradation in the CNS, and penetration of immune cells into the CNS [165]. Interestingly, there are some bacterial taxa that produce anti-inflammatory agents, such as SCFAs. Example taxa are *Faecalibacterium*, *Coprococcus*, *Clostridium XIVa* species [166,167], which can produce butyrate, and *Megamonas*, which can produce acetate and propionate [168]. In the animal model, transplanting feces from depressed patients into mice can produce depression-like behaviors [169]. The cause of depression like behavior development may be attributed to inflammation-based theory of depression as hippocampal levels of IFN-γ and TNF–α were significantly increased in mice which received transplant [170].

In one study, the composition of the fecal microbiome in MDD patients differed significantly increased *Bacteroidetes*, *Proteobacteria*, *Actinobacteria* and fewer *Firmicutes* were observed compared to healthy individuals [171]. The systematic review on the gut microbiota in anxiety and depression pointed out that in patients with depressive disorders, higher pro-inflammatory species and a lower abundance of taxa producing SCFAs are observed [172].

Changes in diet can produce a rapid effect on the human gut microbiota [173]. For example, the ingestion of omega–3 polyunsaturated fatty acid dietary supplements can positively impact the growth of butyrate-producing bacteria in healthy middle-aged volunteers [174]. The depletion of dietary fiber in the diet of mice resulted in intestinal barrier dysfunction by promoting the growth of bacteria that degrade colonic mucus [175]. Based on research on the link between food and changes in the gut microbiota, the common sentence “You are what you eat” takes on a new meaning.

Dietary prebiotics, according to the terminology established during the 6th meeting of the International Scientific Association of Probiotics and Prebiotics in 2008, can be defined as “a selectively fermented ingredient that results in specific changes in the composition and/or activity of the gastrointestinal microbiota, thus conferring benefit(s) on host health” [176]. Edible mushrooms are a source of polysaccharides, naturally occurring substances that exhibit multidirectional biological activity and can be classified as prebiotics. Their activity is affected by its chemical composition such as monosaccharide composition, molecular weight, and the type of glycosidic bond [177].

Edible mushrooms are considered as a potential source of prebiotics due to the presence of polysaccharides such as α-,β-glucans, chitin that meet the conditions of being prebiotics: they are resistant to conditions of the upper intestinal tract (acidity), stable to food processing and fermented by the gut microbiota, which leads to the production of substrates needed for efficient growth of the gut microbiota. Current knowledge about the influence of edible mushrooms on the microbiota and gut health is summarized in Table 4.

The influence of mushroom ingestion on the gut microbiota can be measured by different approaches. There is increasing research on the impact of mushroom administration on the use of animal models, mostly rodents. The tested substance can be powdered fruiting bodies or mycelium of selected mushroom species or an isolated polysaccharide—mostly the one that cannot be hydrolyzed in an acidic environment.

The analyzes comprises evaluation of microbiota alteration after a certain amount of time of mushroom administration—analyzes of compositional complexity of a bacterial community within a site that increases with the number of present species and with the evenness of their relative abundances (Alpha diversity) and highlight taxonomical differences between pairs of samples (Beta diversity) [207]. Methods for the complex analysis of the gut microbiota are based on 16Sr RNA high–throughput sequencing and metabolomics [121]. Another factor, the *Firmicutes*/*Bacteroidetes* ratio (F/B ratio) during may be important biomarkers that prove no negative effect on the gut microbiota [208]. For example, the addition of powdered *A. bisporus* fruiting bodies to mice resulted in a stable F/B ratio during the course of the experiment, indicating that supplementation with *A. bisporus* in the diet did not result in any negative alteration of the intestinal microbiota [195].

Another important factor contributing to the potential mood-elevating activity of medicinal/edible mushrooms is restoring the intestinal barrier; by this means, the levels of so-called tight junction proteins are measured such as ZO-1, claudin-3, and occludin using the Western blot method [209]. Ethanolic extract of *G. lucidum* administration to: C57BL6/J mice with DDS-induced colitis for 14 days that disrupted the intestinal barrier resulted in recovery of intestinal barrier function by increasing the level of junction proteins, the levels of which, after treatment, were at the same level as in the control group [178]. Disruption of the intestinal barrier may result in some diseases such as irritable bowel syndrome (IBS), obesity, non-alcoholic fatty liver disease (NAFLD), type 2 diabetes mellitus [210,211,212,213]. A disrupted intestinal barrier—the core of the hypothesis of “leaky gut”— may be an important factor in the pathophysiology of depression, as the leakage of bacteria into the bloodstream and pro-inflammatory substances such as endotoxins that increase inflammation can increase the odds of depression [214,215].

Interestingly, psychological stress can affect the permeability of the intestinal barrier by the so-called hypothalamic-pituitary-adrenal axis (HPA axis) [216]. Stress can activate the HPA axis, which leads to cortisol production and release from the adrenal cortex in response to adrenocorticotropic hormone (ACTH). Cortisol can increase gut permeability and thus, cause the penetration of bacterial LPS into the bloodstream, which leads to peripheral inflammation.

## 7. Conclusions

The International Society for Nutritional Psychiatry Research pointed out that human nutrition should be taken into account, especially in patients suffering from MDD or anxiety disorders [217]. There are many studies that show the effect of human nutrition on human health. For example, in the study that involved patients with type 2 diabetes divided into two groups: Mediterranean diet with nuts and the control group, the chances of depression in the experimental group were 41% lower compared to the control group [218]. The high content of omega–3 polyunsaturated fatty acids in the Mediterranean diet is believed to be responsible for lowering the risk of depression due to their antioxidant activity, as well as the rich content of polyphenols and reduced intake of red meat [219,220].

**Figure 2:**
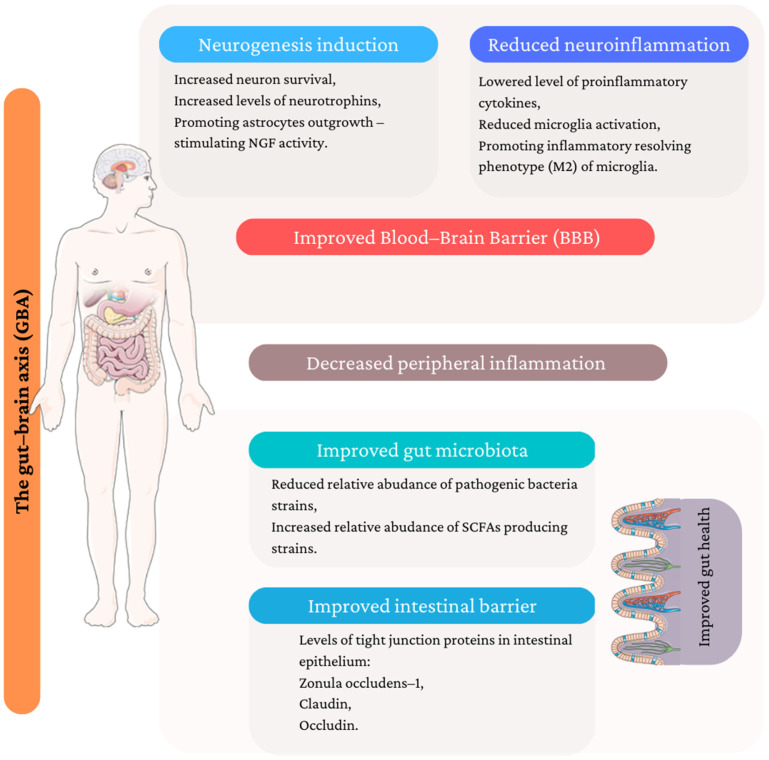
Potential mechanism of antidepressant activity of medicinal/edible mushroom. (Figure composed using Servier Medical Art: http://smart.servier.com/ (accessed on 14 January 2023)).

A plethora of evidence suggests that the introduction of medicinal/edible mushrooms to the daily diet in the form of mushroom-containing dishes and dietary supplements may lower the risk of depression development. The review summarized the potential mechanisms of antidepressant action from different perspectives, which are illustrated in Figure 2.

In conclusion, edible mushrooms should be considered a valuable daily dietary source with potential multidirectional antidepressant activity.

## Figures and Tables

**Table 1:** Content of L–tryptophan, 5–hydroxy–L–tryptophan and tryptamine in fruiting bodies of selected medicinal mushrooms.

Species	Source	L-Tryptophan (mg/100 g dw)	Tryptamine (mg/100 g dw)	5-Hydroxy-L-tryptophan (mg/100 g dw)	Ref.
*Pleurotus citrinopileatus*	Fruiting bodies form commercial cultivation	1.29 ± 0.08	1.29 ± 0.08	128.89 ± 10.67	[39]
*Pleurotus djamor*		24.84 ± 0.97	3.54 ± 0.18	193.95 ± 17.69	
*Pleurotus ostreatus*		5.79 ± 0.06	1.04 ± 0.15	67.45 ± 7.94	
*Auricularia cornea*		0.16 ± 0.050	2.77 ± 0.050	7.35 ± 0.200	[40]
*Armillaria mellea*	Fruiting bodies from natural habitats	4.47 ± 0.01	2.74 ± 0.01	- ^z^	[41]
*Imleria badia*		0.68 ± 0.047	0.18 ± 0.001	- ^z^	
*Boletus edulis*		0.39 ± 0.020	1.17 ± 0.003	0.18 ± 0.001	
*Lactarius deliciosus*		- ^z^	- ^z^	0.25 ± 0.017	
*Hydnum repandum*		0.37 ± 0.09	1.46 ± 1.05	n/a	[42]
*Cantharellus cibarius*		0.01 ± 0.002	0.01 ± 0.002	0.02 ± 0.003	[43]
*Agaricus bisporus*		0.39 ± 0.020	0.06 ± 0.003	- ^z^	
*Sarcodon imbricatus*		13.01 ± 0.01	22.12 ± 0.03	n/a	
*Macrolepiota procera*		3.47 ± 0.050	0.92 ± 0.040	22.94 ± 0.500	[40]
*Suillus bovinus*		25.90 ± 0.200	3.15 ± 0.050	15.83 ± 0.500	
*Tricholoma equestre*		2.851 ± 0.242	2.011 ± 0.141	0.586 ± 0.041	[44]
*Calocera viscosa*		1.26 ± 0.04	n/a	11.88 ± 0.19	

n/a—data not available, ^z^ concentration lower than 0.001 mg/100 g dw.

**Table 2:** Content of selected non-hallucinogenic indole compounds in mycelia of selected edible mushroom.

Species	L-Tryptophan (mg/100 g dw)	Tryptamine (mg/100 g dw)	5-Hydroxy-L-tryptophan (mg/100 g dw)	Ref.
*Pleurotus citrinopileatus*	7.82 ± 0.31	3.71 ± 0.34	368.67 ± 23.53	[39]
*Pleurotus djamor*	24.34 ± 1.44	n/d	703.56 ± 37.79	
*Pleurotus ostreatus*	1.89 ± 0.04	1.03 ± 0.15	120.11 ± 20.12	
*Agaricus bisporus*	14.00 ± 0.300	0.48 ± 0.050	12.50 ± 0.671	[51]
*Sarcodon imbricatus*	1.31 ± 0.6	4.11 ± 0.26	n/a	[52]
*Tricholoma equestre*	1.036 ± 0.093	0.598 ± 0.048	0.344 ± 0.031	[44]
*Imleria badia*	0.827 ± 0.049	0.409 ± 0.045	- ^z^	
*Calocera viscosa*	1.79 ± 0.05	n/a	11.42 ± 0.20	[53]
*Cantharellus cibarius*	0.64 ± 0.013	- ^z^	12.52 ± 0.671	[54]

n/a—data not available, n/d—not detected, ^z^ concentration lower than 0.001 mg/100 g dw.

**Table 3:** Anti-inflammatory activity of selected medicinal/edible mushroom species.

Species	Active Substance	Experience Model	Result	Ref.
*Lentinula edodes*	β-glucan	In vivo: C57BL/6J mice Inflammation: High-fat diet-induced (HFD)	↓Neuroinflammation in hippocampus and prefrontal cortex ↓TNF–α, ↓IL-6, ↓IL-1B in hippocampus) ↓Microglia proliferation and ↓TNF–α, ↓IL-6, ↓IL-1B in PFC	[107]
	Lentinan	In vitro: mice microglia BV–2 cell line Inflammation: LPS induced	↓Microglia inflammation ↓TNF-a, ↓IL-1B ↑IL–10, ↑BDNF Promoted conversion of microglial phenotype from M1 (proinflammatory) to M2 (inflammatory resolving) status	[108]
*Inonotus obliquus*	Lanostane triterpenes: Inonotusols H-N	In vitro: mice microglia BV–2 cell line Inflammation: LPS induced	↓Microglia inflammation ↓NO generation ↓iNOS expression inhibition (the strongest interaction with inonotusols I)	[109]
*Cyathus africanus*	Cyanthane diterpenoids	In vitro: mice microglia BV–2 cell line, PC–12 cell line Inflammation: LPS induced	↓Microglia inflammation ↓NO generation in BV–2 cell line ↑Neurotrophic activity through NGF ↑Neurite outgrowth in PC–12 cell line	[110]
*Auricularia polytricha*	Ethanol and hexane extracts	In vitro: mice microglia BV–2 cell line Inflammation: BPA induced	↓Neuroinflammation by regulation of NG-kB signaling pathway ↑Antioxidant activity ↑SOD-1 enzyme activity	[111]
*Sanguinoderma rugosum*	Mycelial extracts	In vitro: mice hippocampal neuronal HT–22 cell line Inflammation: glutamate induced	↑Cell viability in pretreated with mycelial extract cell line after glutamate inflammation induction The main constituents responsible for the action were: linoleic acid, ergosterol and ethyl linoleate (GC-MS analysis)	[112]
*Hericium erinaceus*	Erinacine A (EA)/*H. erinaceus* extract (HEM)	In vivo: male Sprague-Dawley rats Inflammation: LPS injection intranigrally In vitro: mice microglia BV–2 cell line, CTX TNA2 cell line Inflammation: LPS, IFN-γ induced	In vivo: Both, EA and HEM showed neuroprotective effect on motor dysfunction in rats with LPS-induced neuronal damage. ↓Neuroinflammation ↓TNF-α, ↓IL-1β, ↓iNOS gene expression BDNF levels did not differ from control and experimental groups In vitro: ↓Microglia inflammation ↓NO generation in BV–2 cell line after EA pretreatment ↓iNOS expression in BV–2 cell line after EA pretreatment ↓Astrocytes inflammation ↓TNF-a expression in CTX TNA2 cell line after EA pretreatment The expression levels of IL–1B and iNOS at the same level, regardless whether pretreated with EA or not	[113]
*Grifola frondosa*	o-Orsellinaldehyde	In vitro: primary microglia and astrocytes from mice brain Inflammation: LPS induced	↓Neuroinflammation ↓NO generation ↓iNOS, ↓HO-1 expression ↓NF-κB pathway activation in microglia Promoted conversion of microglial phenotype from M1 (proinflammatory) to M2 (inflammatory resolving) status	[114]
-	Ergothioneine	In vitro: hCMEC/D3 human brain endothelial cell line Inflammation: 7-ketocholesterol induced	↓Neuroinflammation ↓IL-1β, ↓IL-6, ↓IL-8, ↓TNF-α, ↓COX-2 mRNA expression after ergothioneine addition	[115]
*Cordyceps militaris*	*C. militaris* powdered fruiting bodies	In vivo: AD mice Inflammation: intracerebroventricular injection of Aβ_1−42_	↓Neuroinflammation ↓iNOS, ↓COX–2 gene expression ↑Neuronal plasticity ↑BDNF expression	[116]
*Lignosus rhinocerotis*	Lipid rich fraction of the sclerotium	In vitro: mice microglia BV–2 cell line Inflammation: LPS induced	↓Neuroinflammation ↓NO production ↓iNOS, ↓COX–2 gene expression ↑HO–1, ↑NQO–1, ↑Nrf2 gene expression	[117]
	Hot aqueous extract (HAE), an ethanol extract (EE), fractions from the HAE and EE, crude polysaccharides	In vitro: mice microglia BV–2 cell line Inflammation: LPS induced	↓Neuroinflammation 500 μg/mL of HAE → ↓NO production by 88.95% * 250 μg/mL of an n-butanol fraction → ↓NO production by 86.5% * 250 μg/mL of an ethyl acetate fraction of HAE → ↓NO production by 85.93% * * Compared to control group	[118]
*Coriolus versicolor*	*C. versicolor* biomass (mycelium and primordia)	Clinical trial: 40 patients with Meniere’s disease (MD)	↓Oxidation stress in group treated with *Coriolus* supplement ↑Hsp70, ↑HO-1 proteins level in lymphocytes and plasma ↑γ-GC liase activity ↑GSH/GSSG ratio in patients treated with *Coriolus* ↑GSH level in patient treated group	[119]
	*C. versicolor* biomass (mycelium and primordia)	In vivo: male Sprague–Dawley rats Inflammation: no induction	↑Neuroprotection ↑LXA4 level in rat brain, especially in cortex and hippocampus ↑Hsp72, ↑OH–1, ↑thioredoxin proteins level	[120]
*Ganoderma lucidum*	Deacetyl ganoderic acid F (DeGA F)	In vivo: zebrafish and C57BL/6J mice Inflammation: LPS induced In vitro: mice microglia BV–2 cell line Inflammation: LPS induced	In vivo: ↓Inflammation in zebrafish ↓NO production ↓Neuroinflammation in mice ↓TNF-α, ↓IL-6, ↓iNOS, ↓p-Akt, ↓p-IKKα/protein levels DeGA F attenuated LPS–induced cell morphology changes In vitro: ↓Neuroinflammation through NF-κB signaling pathway ↓p65 nuclear protein level and migration reduction ↓p-Akt, ↓p-IKKα/β, ↓p-IκBα expression	[121]
*Armillaria mellea*	Fr.2 fraction (5-hydroxymethylfurfural, vanillic acid, syringate)	In vitro: mice microglia BV–2 cell line Inflammation: LPS induced	↓Neuroinflammation ↓NO, ↓IL–6, ↓IL-1β, ↓TNF-α in a dose depended manner ↓phosphorylation level of NF-κB p65, IκB-α and JNKs pathway	[122]

↓—decrease, ↑—increase.

**Table 4:** Selected medicinal mushroom species effect on gut microbiota.

Medicinal Mushroom Species	Active Ingredient	Experimental Model	Gut Microbiota Alteration in Treated Group	Biological Activity	Ref.
*Ganoderma lucidum*	Ethanolic extract	In vivo: C57BL6/J mice Dextran sulphate sodium administration (DSS)-induced colitis	↑*Turicibacter* ↑*Bifidobacterium* ↑*Parabacteroides* abudance ↓*Escherichia/Shigella* ↓*Bacteroides* ↓*Staphylococcus* abudance	Restoration of the intestinal barrier ↑MUC2, ↑ZO-1, ↑claudin-3, and ↑occludin in DDS-induced colitis group with supplementation of ethanolic extract. Reduction of local bowel inflammation ↓iNOS, ↓COX–2 activity	[178]
	Ethanolic extract of *G. lucidum* mycelium (GLAA)	In vivo: ICR mice, SPF Gut microbiota depleted by antibiotic	↑*Bifidobacteriaceae Bidifobacterium* ↑*Lactobacillaceae* *Lactobacullus* (*L. reuteri*) ↑*Porphyromonadaceae Odoribacter* ↑*Erysipelotrichales* *Turicbacter* ↓*Ruminococcaceae* *Oscillibacter* ↓*Anaerotignum* ↓*Roseburia*	Addition of GLAA reduced required time for sleep induction by 50% in pentobarbital-induced hypnosis model ↑5-HT in the hypothalamus in treated with GLAA group (50 and 100 mg/kg)	[179]
	Hydroalcoholic extracts	In vivo*:* C57BL/6 mice High-cholesterol diet (HCD)	Reversed gut dysbiosis caused by HCD ↑*Lactobacillus* ↓*Bacteroides acidifaciens* ↓*Mucispirillum schaedleri* ↓*Parabacteroides distasonis*	*G. lucidum* extracts revealed to be a novel transcriptome modulator—prevention of metabolic disorders associated with hypercholesterolemia	[180]
	Polysaccharides from hot water extraction	In vivo*:* Sprague-Dawley rats (SPF) HFD and streptozotocin-induced type 2 diabetes mellitus	↑*Blautia* ↑*Dehalobacterium* ↑*Parabacteroides* ↑*Bacteroides* ↓*Aerococcus* ↓*Ruminococcus* ↓*Corynebactrium* ↓*Proteus*	Administration of mushroom polysaccharides showed antidiabetic effect	[181]
*Antrodia cinnamomea*	Exopolysaccharides	In vivo*:* ICR mice Antibiotics induced gut dysbiosis (lincomycin)	↑*Lactobacillus* ↑*Roseburia* ↑*Ligilactobacillus* ↑*Lachnospiraceae_NK4A136* group abudance ↓*Enterococcus Shigella* abudance	Reduced peripheral inflammation ↓IL–6 and ↓TNF-a serum levels compared to control group	[182]
	Isolated polysaccharides	In vivo: Lingnan yellow-heathered female chickens LPS induced liver inflammation	Restrained the decline of beneficial cecal microbiota (typically *Lactobacillus*, *Faecalibacterium*, and *Christensenellaceae R-7* group)	Hepatoprotective effect—reversed LPS-induced liver inflammation ↓TLR4/NF-κB signaling pathway expression in the liver tissues	[183]
*Morchella esculenta*	Isolated polysaccharides	In vivo: BALC/c mice (SPF) HFD	↑*Lactobacillus* ↓*Enterococcus*	Improved intestinal barrier ↑TJs proteins ↓Inflammation in bowel ↓iNOS, ↓COX-2, ↓ TNF-α, and ↓IL-6 overexpression (immunoblotting) ↓TLR4 inflammatory related signaling pathways	[184]
*Lyophyllum decastes*	Isolated polysaccharides	In vivo: C57BL/7J mice HFD	↓*Firmictues/Bacteroidetes* ratio ↑*Lactobacillus johnsonii* *Bacteroides intestinalis*	Antiobestity effect of extracted polysaccharides due to microbiota alteration and secondary bile-acids production activation	[185]
*Wolfiporia cocos*	Including water-soluble polysaccharides (PCX), water-insoluble polysaccharides (PCY), and triterpenoid saponins (PCZ)	In vivo: healthy Kunming mice	The strongest altering gut microbiota activity was found in PCY fraction: ↑*Lactobacillus* In mice fed with PCX: ↑*Deferribacterota*	PCX: ↑IL–10 levels in the liver, spleen tissues and serum, PCY: ↓IFN–γ level in the liver, PCZ: ↓TNF-α level in liver and spleen PCX and PCZ: ↑IFN–γ	[186]
	Triterpenoid fraction	In vivo: Spague–Daley rats Unpredictable mild stress model (CUMS)	Restoration of altered gut microbiota by CUMS: ↓*Firmicutes/Bacteroidetes* ratio	↑BDNF, ↑NGF in the hippocampus	[187]
*Auricularia polytricha*	Freeze dried ethanolic extracts of fruiting bodies	In vivo: ICR mice (SPF) DSS-induced intestinal dysbiosis	↑*Ruminococcaceae* ↑*Lachnospiraceae* ↑*Prevotellaceae* ↑*Bifidobacteriaceae* ↑*Erysipelotrichaceae*	Anti-inflammatory activity: ↓F-κB and MAPK/ ERK1/2 signaling pathway ↑Keap1/Nrf2 signaling pathways	[111]
*Flammulina velutipes*	Freeze dried ethanolic extracts of fruiting bodies				
*Pleurotus citrinopileatus*	Isolated Polysaccharide–peptide I (PSI),Polysaccharide–peptide II (PSII)	In vitro batch culture*:* fecal samples from healthy human volunteers on YCFA medium	PSI: ↓*Escherichia-Schigella* PSII: ↑*Bifidobacterium* ↑*Lactococcus* ↑*Lactobacillus* ↑*Desulfovibrionaceae* ↑*Lachnospiraceae* ↑*Odoribacter* ↑*Coriobacteriaceae* ↑*Blautia* ↓*Escherichia-Schigella*	Administration of PSI resulted in higher production of SCFAs	[188]
*Pleurotus eryngii*	Water soluble polysaccharide fraction	In vivo: C57BL/6J mice HFD	↑*Anaerostipes* ↑*Clostridium* ↑*Lactococcus* ↓*Roseburia* ↓*Lactobacillus*		[189]
	Powdered fruiting bodies	In vivo*:* CD–1 mice DSS-induced colitis	↑*Odoribacteraceae* ↑*Adlercreutzia* ↑*Akkermanisa* ↑*Lactobacillus* ↑*Anaerostipes* ↑*Allobaculum* ↓*Actinobacteria* ↓*Mollicutes* ↓*Desulfovibrionace* ↓*Enterococcaceae* ↓*Turicibacter* ↓*Dorea* ↓*Bacteroides* ↓*Prevotella*	↑Production of SFCAs in dose-depended manner ↓Local—colon inflammation ↓IL-1β, ↓IL-17 No effect on IL–2, IL–17A, IFN–γ,	[190]
*Pleurotus ostreatus*	Powdered fruiting bodies	In vivo: C57BL/6J mice HFD-induced obesity	↑*Oscillospira* ↑*Lactobacillus* group ↑*Bifidobacterium* ↑*Anaerostipes* ↑*Anaerovorax* ↑*Anaerofustis* ↑*Ruminococcus* ↑*Coprococcus* ↓*Bacteroides* ↓*Roseburia* ↓*Acinetobacter* ↓*Agrobacterium* ↓*Microbacterium* ↓*Novosphingobium* ↓*Streptococcus* ↓*Prevotella* ↓*Sphingomonas* ↓*Macrococcus* ↓*Lactococcus* Compared to HFD only		[191]
	Powdered fruiting bodies	In vivo: piglets (crossbred: Duroc × Large white × Landrace)	↑*Prevotella* ↑*Anaerovibrio* ↑*Veillonellaceae*	↑Production of SFCAs in group with supplementation of *P. ostreatus*,	[192]
*Pleurotus sajor-caju*	Powdered mycelium	In vivo: Zucker rats	↑*Faecalibaculum* ↑*Bifidobacterium* ↑*Roseburia* ↑*Blautia* ↓*Escherichia-Shigella*	↑Production of SCFAs in the colon	[193]
*Agaricus bisporus*	Roasted fruiting bodies	Open label crossover study in healthy adults (*n* = 32)	↑*Bacteroides* ↑*Parabacteroides* ↑*Coprococcus* ↑*Sutterella* ↑*Anaerostipes* Comparing to diet with meat		[194]
*Agaricus bisporus*	Powdered fruiting bodies	In vivo: C57BL/J mice	Stable Firmicutes/Bacteroidetes ratio ↑*Verrucomicrobia* ↑*Akkermansiaceae* ↑*Tannerellaceae* ↓*Prevotellaceae*	↓Inflammation related genes levels such as IL–6, Nox–2 (Portobello only), Hmox–1	[195]
*Agaricus bisporus brunnescens*					
*Armillaria mellea*	*A. mellea* fermentation liquor	In vivo: Sprague–Dawley rats Insomnia model by the p-chlorophenylalanine (PCPA) induction	↑*Lachnospiraceae NK4A136* group ↑*Lachnospiraceae* ↑the *[Eubacterium] xylanophilum* group ↑*Ruminococcus* ↑*Candidatus* ↑*Saccharimonas* ↑the *[Eubacterium] coprostanoligenes* group ↓*Lactobacillus* ↓*Maribaculum* ↓*Prevotellaceae UCG-001*	Administration of AFL resulted in ↑5-HT_1A_ and 5-HT_2A_ proteins in hippocampus comparing to model group ↓Peripherial inflammation reduced after AFL administration in dose-dependent manner ↓IL-6, ↓TNF–α, ↓IL–1β	[196]
*Lentinula edodes*	Isolated polysaccharide—lentinan	In vivo: C57BL/6J mice, HFD	↑*Actinobacteria* ↑*Firmicutes* ↑*Bifidobacterium* ↓*Proteobacteria* ↓*Epsilonbacteraeota*	Addition of lentinan to HD diet resulted in improved intestinal barrier ↑TJs proteins (occludin, ZO–1) at mRNA and protein level Improved glucose tolerance	[197]
	Isolated β–glucan	In vivo: C57BL/6J mice HFD	↑*Clostridiales* ↑*Lachnospiraceae* ↑*Ruminococcaceae*	Addition of lentinan to HFD resulted in improved intestinal barrier ↑TJs proteins (occludin) at protein level ↓Serum LPS → ↓peripheral inflammation	[107]
	Polysaccharides	In vivo*:* C57BL/6J male mice DSS-induced colitis	In DDS-induced group: ↑*Bacteroides* ↑*Helicobacter* ↑*Parasutterella* ↓*Firmicutes* Addition of FMP to diet reversed negative gut microbiota alteration	Improved intestinal barrier after mushroom addition to diet: ↑TJs proteins (occludin, ZO–1) level Local inflammation in colon ↓TNF–α, ↓IL–1β, ↓IL–6 ↑Production of SCFAs	[198]
*Hericium erinaceus*	Powderized *H. erinaceus* fruting bodies	In vivo: 11-years old dogs	↑*Bacteroidetes* ↑*Bacteroidales* ↓*Firmicutes* ↓*Streptococcus* ↓*Tyzzerella* ↓*Campylobacteraceae* ↓*Campylocaber*		[199]
	Powdered fruiting bodies(as dietary supplement)	A pilot study with 13 healthy adults	↑*Roseburia faecis* ↑*Faecalibacterium prausnitzii* ↑*Eubacterium rectale* ↑*Fusicatenibacter saccharivorans* ↑*Kineothrix alysoides* ↑*Gemmiger formicilis* ↑*Dorea longicatena* ↓*Streptococcus thermophilus* ↓*Roseburia intestinalis* ↓*Bacteroides caccae* ↓*Anaerostipes hadrus*	Short supplementation (7 days) resulted in increased alpha and beta diversity—it may produce short term effect retention.	[200]
*Dictyophora indusiata*	Different fractions of polysaccharides: soluble (DIPX) and insoluble in water (DIPY)	In vivo*:* Kunming mice	In DIPY group: ↓*Lachnospiraceae* ↑*Lactobacillus* Gut microbiota composition in DIPX group did not differ to the control group	↑Production of SCFAs in DIPY group Production of SCFAs in DIPX groups did not differ comparing to the control group	[201]
	Isolated polysaccharide	In vivo*:* BALB/c mice HFD	Addition of polysaccharide resulted in reversion of HFD-induced gut alteration: ↓*Firmicutes/Bacteroidetes* ratio	Improved intestinal barrier after polysaccharide supplementation to diet: ↑claudin-1, ↑occludin, and ↑zonula occludens (ZO-1) ↓Serum LPS → ↓peripheral inflammation	[202]
*Helvella leucopus*	Isolated polysaccharide (p-HLP)	In vivo: C57BL/6 DSS-induced colitis	↑*Bacteroidaceae* ↑*Prevotellaceae* ↑*Akkermansiaceae*	↓Local inflammation ↓IL–6, ↓IL–1b, ↓TNF–a, ↓iNOS, ↓COX–2 at mRNA level ↑IL–10 at mRNA level	[203]
*Tremella fuciformis*	Isolated polysaccharides	In vivo*:* C57BL/6 DSS-induced colitis	↑*Lactobacillaceae* ↑*Lactobacillus* ↑*Marinifilaceae* ↓*Helicobacter* ↓*Ruminococcaceae*	↓ Local inflammation ↓IFN-γ, ↓IL-1β, ↓IL-6, ↓TGF-β, ↓TNF-α ↑IL–10 ↑PPAR-γ activity	[204]
*Grifola frondosa*	Isolated polysaccharides	In vivo: Wistar rats HFD	↑*Helicobacter* ↑*Intestinimonas* ↑*Barnesiella* ↑*Parasutterella* ↑*Ruminococcus* ↑*Flavonifracter* ↓*Clostridium-XVIII* ↓*Turicibacter*		[205]
	Isolated heteropolysaccharide (GFP-N)	In vivo*:* ICR mice T2DM induced	↑*Porphyromonas gingivalis* ↑*Akkermansia muciniphila* ↑*Lactobacillus acidophilus* ↑*Tannerella forsythia* ↑*Bacteroides acidifaciens* ↑*Roseburia intestinalis*	Improved oral glucose test, alleviated insulin resistance, decreased the fast blood glucose level	[206]

↓—decrease, ↑—increase.

## Data Availability

Data is contained within the article.

## Abbreviations

World Health Organization (WHO), tumor necrosis factor-alpha (TNF–α), interferon gamma (INF–γ), monoamine oxidase (MAO), selective serotonin reuptake inhibitors (SSRIs), brain-derived neurotrophic factor (BDNF), Major Depressive Disorder (MDD), neurotrophin–3 (NT–3), neurotrophin–4 (NT–4), nerve growth factor (NGF), L–tryptophan (Trp), rapid acting antidepressant (RAAD), treatment–resistant depression (TRD), 5-hydroxy–L–tryptophan (5-OH-L-Trp), Trp hydroxylase (TPH), large neutral amino acids (LNAAs), blood-brain barrier (BBB), colon epithelial cells (CaCo-2), rapid-acting antidepressants (RAADs), medial prefrontal cortex (mPFC), high–fat diet (HFD), erinacine A (EA), *H. erinaceus* extract (HEM), hot aqueous extract (HAE), ethanol extract (EE), Meniere’s disease (MD), deacetyl ganoderic acid F (DeGA F), toll–like receptors (TLRs), pathogen–associated molecular patterns (PAMPs), danger-associated molecular patterns (DAMPs), lipopolysaccharide (LPS), nuclear factor kappa-light-chain-enhancer of activated B cells (NF-κB), inducible nitric oxide synthase (iNOS), nitric oxide (NO), cyclooxygenase–2 (COX-2), p75 neurotrophin receptor (p75^NTR^), tyrosine kinase receptor (Trk), polysaccharide–peptide (PGL), tropomyosin receptor kinase B (TrkB), synaptophysin (SYP), Center for Epidemiologic Studies Depression Scale (CES-D), Indefinite Complaints Index (ICI), 2α-hydroxy-inotodiol (2α-HI), cuprizone (CPZ), short-chain fatty acids (SCFAs), dextran sulphate sodium administration (DSS), ethanolic extract of *G. lucidum* mycelium (GLAA), high-cholesterol diet (HCD), Specific free-pathogen (SFC), water-soluble polysaccha-rides (PCX), water-insoluble polysaccharides (PCY), triterpenoid saponins (PCZ), unpredictable mild stress model (CUMS), polysaccharide–peptide I (PSI), polysaccharide–peptide II (PSII), p-chlorophenylalanine (PCPA), alkaline phosphatase (ALP), different fractions of polysaccharides: soluble in water (DIPX), different fractions of polysaccharides: insoluble in water (DIPY), zonula occludens (ZO-1), irritable bowel syndrome (IBS), non-alcoholic fatty liver disease (NAFLD), hypothalamic-pituitary-adrenal axis (HPA axis), adrenocorticotropic hormone (ACTH).
